# VZV reactivation, immune remodeling, and dementia risk

**DOI:** 10.3389/fimmu.2026.1953013

**Published:** 2026-10-14

**Authors:** Lijun Zhang, Shuang Wu, Yaru Quan, Wenrong Yao

**Affiliations:** 1 Beijing ABZYMO Biosciences Co., Ltd., Beijing, China; 2 Jiangsu Recbio Technology Co., Ltd. Taizhou, China; 3 State Key Laboratory of Drug Regulatory Science (SKLDRS), National Institutes for Drug Control, Beijing, China

**Keywords:** dementia, neuroprotection, recombinant zoster vaccine, trained immunity, Varicella-Zoster virus (VZV)

## Abstract

Varicella-Zoster Virus (VZV) is a highly dermatotropic and neurotropic α-herpesvirus that causes varicella (chickenpox) during primary infection, subsequently establishing lifelong latency, and reactivates to cause herpes zoster when host immunity declines or suppressed. Recent large-scale epidemiological studies consistently associate herpes zoster immunization, particularly with the recombinant zoster vaccine (RZV), with a significantly reduced risk of incident dementia. These findings have prompted a rigorous re-evaluation of the infectious etiology of progressive neurodegeneration. This review integrates the intersecting molecular mechanisms of VZV latency, localized ganglionic immunosenescence, and systemic inflammaging. We critically evaluate a synergistic VZV-HSV-1 amyloidogenic cascade, while explicitly addressing the biological limitations of extrapolating these transient interactions to human central nervous system (CNS) neuropathology. Furthermore, we propose the hypothesis that AS01B-induced trained immunity may contribute to neuroprotection. Therefore, this review provides insight for understanding the systemic consequences of chronic VZV infection and the impact of adjuvant-mediated immune remodeling in aging populations.

## 1 Introduction

Varicella-zoster virus (VZV) is a highly evolved, human-restricted neurotropic α-herpesvirus that establishes lifelong latency in sensory neurons following primary infection (1). Traditional clinical paradigms have long simplified VZV infection into a strict biphasic event: primary infection causing childhood varicella followed by ganglionic latency and subsequent reactivation as herpes zoster (HZ) upon declining host immunity (2, 3). However, global epidemiological registries reveal a profound shift: while childhood varicella-preventative immunization programs have reduced pediatric varicella incidence, the absolute morbidity and healthcare burden of herpes zoster and its severe neurological sequelae (such as postherpetic neuralgia and vasculopathy) are rising sharply in populations aged over 50 years (2–5).

High-resolution single-cell transcriptomics, structural biology, and large-scale electronic health record (EHR) databases have prompted a paradigm shift in our understanding of chronic VZV pathogenesis. Emerging analyses indicate that VZV is not merely a superficial dermatological pathogen causing self-limiting vesicular eruptions; rather, it represents a chronic systemic burden that drives persistent immuno-metabolic exhaustion (6). Type 2 diabetes (T2D) and metabolic syndrome can weaken VZV-specific cell-mediated immunity and increase baseline systemic inflammation, making viral reactivation more likely (7–9). Conversely, clinical and translational evidence links subclinical or recurrent VZV reactivation to chronic degenerative and cerebrovascular conditions, including Alzheimer’s disease (AD) and cerebral amyloid angiopathy (CAA) (10, 11).

Natural experiments and propensity-score-matched cohort studies show that older adults receiving the live-attenuated zoster vaccine (ZVL) exhibit an approximate 20% relative reduction in incident dementia diagnoses (12). Furthermore, the transition to the recombinant vaccine (RZV) is associated with a 17% increase in dementia-free survival time (translating to 164 additional days lived without a diagnosis) compared to ZVL (13). Strikingly, cohorts immunized with the respiratory syncytial virus (RSV) vaccine, which utilizes the dose-sparing AS01E liposomal formulation (14, 15) from the same AS01 adjuvant family, demonstrated a comparable 29% reduction in dementia risk (16). Because RSV is a non-neurotropic respiratory pathogen, this finding supports the hypothesis that adjuvant-associated immune remodeling may contribute to neuroprotection independently of the vaccine antigen. Such an effect could complement the pathogen-specific suppression of viral reactivation (16). This review reassesses the systemic pathogenic effects of VZV infection and examines how adjuvant-induced immune responses and trained immunity might modify neurodegenerative processes.

## 2 VZV biology and immunology

VZV is a globally ubiquitous human α-herpesvirus responsible for two distinct clinical entities: primary infection manifests as varicella (chickenpox), whereas viral reactivation from latency precipitates HZ (shingles). VZV exhibits high transmissibility, disseminating primarily via aerosolized respiratory droplets and direct contact with infectious vesicular lesions, with efficient viral replication and shedding in the skin serving as critical determinants for viral transmission. The epidemiological footprint of VZV varies regionally, largely contingent upon population-level immunity. Global Burden of Disease (GBD) studies from 1990 to 2019 highlight a substantial decline in age-standardized varicella incidence among children (<20 years), juxtaposed with a marked increase in HZ incidence among older adults (>50 years). The risk of developing HZ escalates precipitously with advancing age, with the disease burden accelerating particularly rapidly in individuals over 70.

VZV is an enveloped α-herpesvirus with a 150–200 nm diameter and a 125-kbp linear double-stranded DNA genome encoding at least 70 open reading frames (ORFs) (17). Viral entry and cell-to-cell spread require nine envelope glycoproteins. Specifically, the abundant envelope glycoprotein E (gE) binds to host insulin-degrading enzyme (IDE) via its unique N-terminal domain (residues 1-188) (18, 19), while other viral glycoproteins engage the mannose-6-phosphate receptor (M6PR) to facilitate viral transport and cellular entry (20).

Following primary infection in the tonsillar crypts, VZV exhibits marked tropism for activated memory T cells expressing skin-homing markers, utilizing these cells to bypass the endothelial barrier and seed cutaneous lesions (21, 22). Concurrently, retrograde axonal transport delivers the viral genome from active cutaneous lesions to the nuclei of sensory neurons within the dorsal root ganglia (DRG) and trigeminal ganglia (TG), where a covert latency reservoir is established (23, 24). Notably, the absence of documented varicella does not exclude latent VZV infection. The live-attenuated Oka vaccine strain can establish latency in neuronal tissue and reactivate (25).

However, modern molecular virology has dismantled the paradigm of a transcriptionally silent VZV latency, exposing a dynamic transcriptional and immunological landscape (26). The older models proposed that VZV latency is maintained by the spatial sequestration of immediate-early (IE) transcription within the host cell cytoplasm, ultra-deep sequencing of human trigeminal ganglia has shown that latent VZV transcription is highly restricted, predominantly producing the VZV latency-associated transcript (VLT) (27, 28). VLT is encoded antisense to the major viral transactivate ORF61 and specifically suppresses ORF61 gene expression to maintain genome dormancy (26). VLT functions as a core regulatory element maintaining genome dormancy. During subclinical transitions toward lytic reactivation, alternative splicing generates VLT-ORF63 fusion transcripts, which encode a novel fusion protein (pVLT-ORF63) capable of inducing broad viral gene transcription (27, 28). These transient transcriptional fluctuations do not cause macroscopic herpetic lesions but drive the intermittent leakage of viral antigens into the local ganglionic microenvironment, necessitating continuous immunosurveillance by host T cells to maintain systemic dormancy (28).

To suppress this antigen leakage, the host must maintain a specialized garrison of tissue-resident memory T (TRM) cells. Immunological profiling reveals that these TRM cells—predominantly CD8+—tightly encircle infected neurons and satellite glial cells, enforcing viral dormancy via non-cytolytic mechanisms such as localized IFN-γ secretion (29). This continuous immunological engagement extracts a metabolic “latency tax.” With advancing age and persistent antigen exposure, these localized T cell pools undergo clonal exhaustion (30), accumulating senescent phenotypes defined by high expression of the inhibitory receptor PD-1 (31, 32). This localized immune-metabolic decompensation permits IE proteins to translocate to the nucleus, catalyzing viral reactivation (**Figure 1**).

**Figure 1:**
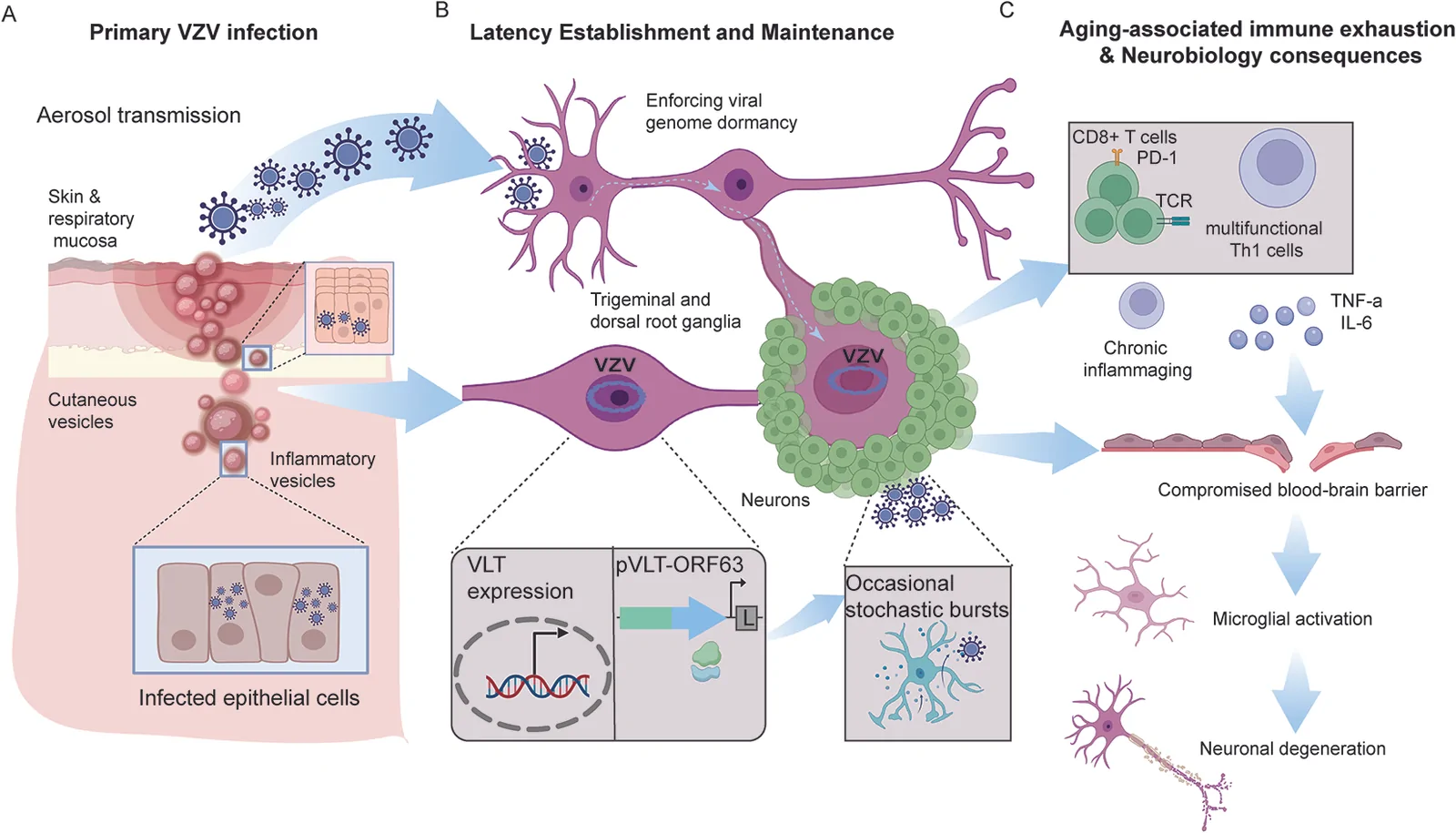
The PNS-to-CNS pathogenic axis of Varicella-Zoster Virus **(VZV)** latency, reactivation, and indirect neurodegeneration. **(A)** Primary VZV infection is initiated via aerosol transmission through the skin and respiratory mucosa, precipitating viral replication within infected epithelial cells and the subsequent formation of cutaneous inflammatory vesicles. **(B)** Latency establishment and maintenance occur following the resolution of acute infection, wherein the virus undergoes retrograde axonal transport along sensory nerve fibers to establish a lifelong covert reservoir within the nuclei of sensory neurons located inside the trigeminal and dorsal root ganglia. Within these niches, viral genome dormancy is strictly enforced via the constitutive expression of the antisense VZV latency-associated transcript (VLT). **(C)** Aging-associated immune exhaustion and neurobiological consequences. With advancing host age, declining cell-mediated immunity drives ganglionic CD8+T and Th1 cells into an exhausted phenotype characterized by PD-1 upregulation, compromising viral containment. This localized immune decompensation triggers a transcriptional switch from dormancy-maintaining VLT expression to the alternative splicing of the pVLT-ORF63 fusion transcript, permitting occasional stochastic bursts of subclinical reactivation. The resulting antigen leakage fuels chronic inflammaging via elevated TNF-α and IL-6 secretion, which disrupts endothelial tight junctions and compromises blood-brain barrier integrity. The subsequent influx of peripheral neurotoxic factors incites persistent microglial activation, ultimately culminating in progressive neuronal degeneration. Figure created with BioRender.com under license.

## 3 VZV pathogenesis and molecular mechanisms of immune evasion

As a neurotropic herpesvirus, the VZV life cycle is characterized by an extensive and intricate arms race with the host immune network, culminating in a molecularly exquisite suite of immune evasion strategies forged over millions of years of co-evolution. During the nascent stages of infection, VZV rapidly deploys early proteins to paralyze the host’s antiviral sensor arrays. Mechanistically, VZV surface glycoproteins actively induce mitophagy, directing the physical degradation of mitochondrial antiviral signaling protein (MAVS) on the outer mitochondrial membrane and the STING complex on the endoplasmic reticulum (33–35). This preemptive strike effectively uncouples the RIG-I/MDA5 and cGAS-STING pathways, profoundly abrogating type I interferon (IFN) induction (34). Concurrently, the VZV-encoded ORF61 protein functions as a viral E3 ubiquitin ligase mimic. By specifically binding and stabilizing the NF-κB inhibitor IκBα, ORF61 prevents its proteasomal degradation, thereby arresting the nuclear translocation of NF-κB and silencing the downstream transcription of pro-inflammatory cytokines (36).

Systemic dissemination of VZV relies heavily on cell-associated viremia. Upon infecting memory T cells in the tonsils, the virus hijacks cutaneous homing receptors (CLA and CCR4) expressed on the T cell surface to facilitate targeted transit. However, if infected T cells undergo unrestrained viral-induced fusion, they would form massive multinucleated syncytia, thereby losing the structural capacity to traverse the capillary endothelium and penetrate cutaneous tissues (21)- (37).To facilitate systemic transit without triggering unchecked cell fusion (syncytia), VZV utilizes an immunoreceptor tyrosine-based inhibition motif (ITIM) on the cytoplasmic tail of glycoprotein B (gBcyt) (38). Host kinases phosphorylate the central tyrosine residue (Y881), locking the gB/gH-gL fusion machinery in an auto-inhibited state, allowing the virus to transport silently within mononuclear T cells (39).This underscores the gBcyt ITIM as an evolutionary masterstroke, precisely titrating the balance between viral dissemination and tissue destruction.

To subvert the host adaptive humoral immunity, VZV employs an exceptionally deceptive strategy termed “antibody bipolar bridging” (ABB). Envelope glycoproteins gE and gI co-assemble in the endoplasmic reticulum to form a heterodimeric complex that structurally and functionally mimics host IgG Fc receptors (vFcR) (18). During a standard antiviral immune response, the Fab regions of host-derived specific neutralizing IgGs engage viral surface targets. However, on the surface of VZV-infected cells, unbound gE/gI complexes rapidly capture the Fc regions of these antigen-bound antibodies, creating an “antigen-Fab-antibody-Fc-vFcR” bipolar bridged configuration (40). This steric entrapment effectively masks the Fc domain, extinguishing recognition by Fcγ receptors on macrophages and NK cells. Consequently, essential humoral effector functions, including antibody-dependent cellular cytotoxicity (ADCC) and antibody-dependent cellular phagocytosis (ADCP), are robustly neutralized. This molecular blockade empowers the virus to sustain cell-to-cell spread even in environments saturated with high-titer neutralizing antibodies.

## 4 VZV-HSV synergistic pathogenesis and proposed mechanism hypotheses of the amyloid cascade

Direct VZV infection of cerebral arteries induces a severe vasculopathy characterized by persistent vascular remodeling and matrix metalloproteinases (MMPs) upregulation, which compromises blood-brain barrier integrity (41, 42). Transcriptomic and proteomic interrogation reveals that VZV infection of primary human brain vascular adventitial fibroblasts (HBVAFs) induces an epigenetic and metabolic reprogramming of the host transcriptome, marked by the highly specific upregulation of amyloid precursor protein (APP) and islet amyloid polypeptide (IAPP, or amylin) (43). Under physiological conditions, amylin is principally synthesized by pancreatic β-cells to modulate systemic glycemic tone. However, within VZV-infected microvascular niches, massive ectopic amylin expression strictly co-deposits with Aβ (10, 44).This virus-catalyzed protein misfolding cascade physically compromises the BBB and drives pancreatic β-cell exhaustion and apoptosis. Consequently, this provides a biophysical link to the clinical observation that VZV-infected cohorts exhibit an increased risk of precipitous glycemic deterioration and metabolic syndrome (45, 46).

As a paradigm of neurodegenerative disorders, Alzheimer’s disease (AD) is supported by a growing body of reported data that warrant exploring the correlation between varicella-zoster virus (VZV) infection-induced neuroinflammation and AD risk. Clinical data reveal elevated amyloid and amylin levels in the cerebrospinal fluid (CSF) of patients with VZV vasculopathy, correlates with high anti-VZV antibody titers (9). *In vitro* models using primary human spinal astrocytes and brain vascular adventitial fibroblasts suggest that VZV infection has correlated with the intracellular production and accumulation of both Aβ and amylin (43, 46). Mechanistically, host insulin-degrading enzyme (IDE) serves as a functional receptor for VZV, wherein the VZV surface glycoprotein gE directly binds to IDE via its N-terminal domain (amino acids 1–188). As IDE functions as a primary protease for soluble amyloid-beta clearance, its steric sequestration by VZV gE provides a biophysical rationale for diminished amyloid clearance during viral replication (18, 19). Concurrently, within the cerebrovascular endothelium, cleaved fragments of VZV glycoprotein B function as catalytic “structural templates” in the extracellular matrix, directly accelerating the kinetic assembly of ectopic amylin and Aβ42 monomers into neurotoxic fibrils (44). Furthermore, epidemiological studies have reinforced this link, demonstrating that VZV reactivation (herpes zoster) is associated with an increased long-term risk of dementia, while vaccination against herpes zoster is associated with a reduced risk, likely by limiting CNS damage and the associated inflammatory processes (6, 12, 13, 47).

VZV and HSV-1 are phylogenetically grouped within the α-herpesvirus subfamily, sharing pronounced neurotropism and establishing lifelong latency within overlapping ganglionic niches (48). In engineered 3D human brain-like tissue models, acute VZV infection of neural networks harboring quiescent HSV-1 disrupts its transcriptional silencing, initiating lytic reactivation associated with robust amyloid-beta secretion and pathological Tau hyperphosphorylation (48). However, extrapolating these transient *in vitro* synergistic cascades to the decades-long pathogenesis of clinical Alzheimer’s disease (AD) requires caution. These reductionist constructs lack functional vascular architecture, blood-brain barrier (BBB) dynamics, and microglial complexity. Furthermore, the “pathogen hypothesis” faces significant domain controversy (49). Multiple independent, large-scale postmortem RNA and DNA sequencing analyses have failed to detect enriched VZV or HSV-1 genomic or transcriptomic signatures within the brain parenchyma of confirmed AD patients compared to controls (49, 50). This conflicting genomic evidence suggests an alternative paradigm: rather than acting as a persistent, direct physical driver of parenchymal amyloid plaque formation, VZV may function primarily as an upstream systemic inflammatory trigger. Its reactivation drives chronic immune-metabolic exhaustion and cerebrovascular endothelioses, indirectly accelerating the neurodegenerative cascade of AD (9).

## 5 Mechanistic cross-validation of epidemiology and engineered immunity utilizing real-world clinical data

Dementia currently affects over 55 million people globally, a figure projected to triple by 2050. In **Figure 2**, evidence from five core studies published between 2024 and 2025 consistently demonstrates that vaccination against varicella-zoster virus (VZV) is associated with a reduced risk of dementia (12, 13, 16, 51, 52). These studies nevertheless estimate different quantities, including relative risks (RRs), absolute risk differences and restricted mean time lost (RMTL) ratios (**Figure 2A, B**). In a multicenter, propensity-score-matched cohort analysis of the US Optum database, researchers demonstrated that ZVL recipients had lower cumulative-hazard RRs for dementia than pneumococcal polysaccharide vaccine (PPSV23) at three years (RR = 0.67, 95% CI: 0.65–0.70) and five years (RR = 0.73, 95% CI: 0.70–0.75). For recipients of at least two doses of recombinant zoster vaccine (RZV), the corresponding RRs vs PPSV23 were 0.73 (95% CI: 0.66–0.80) and 0.83 (95% CI: 0.74–0.94) (52). Within the RZV-treated population, at least two doses were associated with lower dementia risk than one dose at three years (RR = 0.76; 95% CI, 0.71–0.82) and five years (RR = 0.81; 95% CI, 0.74–0.88). In the head-to-head comparison with ZVL, the estimate for at least two RZV doses was not significant at three years (RR = 0.89; 95% CI, 0.74–1.08), but was lower at five years (RR = 0.82; 95% CI, 0.69–0.98). Taquet et al. used the rapid US transition from ZVL to RZV as a separate natural experiment. In matched cohorts of 103,837 individuals per group, RZV versus ZVL was associated with a dementia RMTL ratio of 0.83 (95% CI, 0.79–0.87) and 164 additional diagnosis-free days among affected individuals. The HZ RMTL ratio was 0.65 (0.61–0.69). HZ estimates were similar in females and males, whereas the dementia association was larger in females (interaction P = 0.017). Together, these results support a graded association across vaccine exposure, dose completion and dementia outcome, but they do not directly measure immune protection.

**Figure 2:**
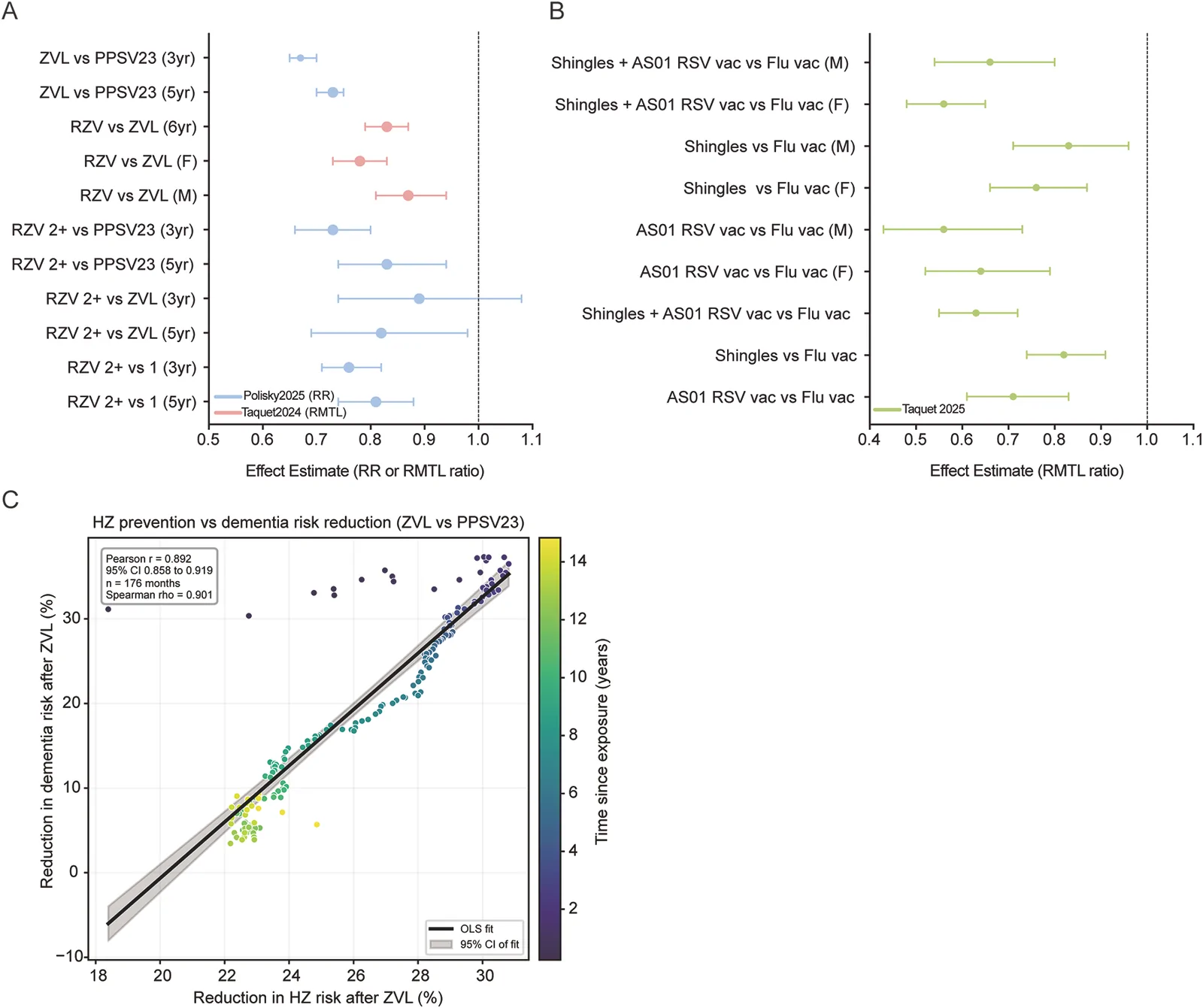
VZV vaccination, AS01-adjuvanted vaccination and dementia outcomes. **(A)** Dementia estimates from Polisky et al. (2025) (52) and Taquet et al. (2024) (13). Polisky values are cumulative-hazard RRs at three or five years; Taquet values are RMTL ratios over up to six years. These statistics share a null value of 1 but should not be pooled or treated as interchangeable. Horizontal bars denote 95% CIs. **(B)** Eighteen-month dementia RMTL ratios from Taquet et al. (2025) (16) for RSV vaccination, shingles vaccination or both vaccines versus influenza vaccination. **(C)** Exploratory concordance between 176 paired monthly reductions in HZ and dementia risk for ZVL versus PPSV23 during months 3–178 from Polisky et al. (2025) (52). Pearson’s product–moment coefficient, computed from the covariance standardized by the two series’ standard deviations, measured the strength and direction of linear association. Spearman’s rank coefficient, computed as the Pearson correlation between the within-series ranks, measured monotonic association without requiring linearity. Both coefficients range from −1 to +1; these positive values indicate that months with larger reductions in HZ risk generally coincided with larger reductions in dementia risk. The panel presents population-level trajectory concordance based on cumulative monthly estimate. These time-series estimates are highly autocorrelated and the conventional Fisher-transformed 95% CI for Pearson’s r was 0.858–0.919, with a nominal two-sided P value of 4.78e-62 for the null hypothesis r = 0. The findings should be treated as an exploratory illustration of aggregate trajectory alignment.

Furthermore, to mitigate residual confounding typical of observational studies—such as the “healthy vaccinee bias,” wherein vaccinated individuals inherently exhibit healthier lifestyles and proactive healthcare-seeking behaviors—other studies utilized natural experimental designs (12). For instance, a regression discontinuity analysis leveraging the SAIL databank in Wales used a strict birthdate eligibility threshold of September 2, 1933, which established demographically similar recipient and non-recipient pools, virtually eliminating confounding factors such as antiviral drug usage, comorbidities, and education level, that have weakened previous investigations (53–55). The initial study estimated that receiving the live-attenuated zoster vaccine decreased the absolute probability of a new dementia diagnosis by 3.5%, representing a 20% relative risk reduction over a 7-year follow-up (12). A subsequent study examined outcomes at different stages of neurodegenerative disease (56). Vaccination was associated with a 3.1% reduction in incident mild cognitive impairment among cognitively healthy individuals and a 29.5% reduction in dementia-specific mortality among adults with established dementia. This protective association was replicated in Australia, where birthdate-based eligibility decreased the absolute probability of new-onset dementia by 1.8% over 7.4 years, and in Ontario, Canada, where zoster immunization led to a 2.0% absolute reduction in dementia diagnoses over 5.5 years (47). Although, the results remain dependent on the validity of the eligibility threshold and outcome ascertainment and do not identify a biological mechanism, also show herpes zoster vaccine may influence outcomes at both early and advanced stages of dementia.

Longitudinal assessments show that the protective effect wanes over time. As post-immunization progress, the relative risk of dementia among ZVL recipients increases, mirroring the clinical efficacy decay curve of the vaccine against herpes zoster reactivation (Pearson’s R = 0.59, 95% CI: [0.30-0.81]; **Figure 2C**) (52). Restricting the exploratory analysis to months 3–178 increased Pearson’s r to 0.892 and Spearman’s ρ to 0.901. However, the coefficient was sensitive to the selected window, first-difference correlations were weak, and both cumulative-hazard trajectories were highly autocorrelated. Conventional confidence intervals and P values are therefore anti-conservative. The observed temporal correspondence indicates exploratory aggregate-level trajectory concordance rather than evidence of individual-level causal mediation or direct biological proof. The dementia RR also approached 1 while the HZ RR remained below 1, which is more consistent with delayed diagnosis than permanent prevention. This temporal alignment is consistent with a biological link between preventing subclinical viral reactivation and mitigating progressive cognitive decline.

Two non-mutually exclusive mechanismic hypotheses have been proposed to explain this vaccine-associated neuroprotection. The first model (Direct Reactivation Suppression) posits that shingles vaccination, by preventing VZV reactivation, blocks downstream vasculopathy, neuroinflammation, and VZV-catalyzed amyloid deposition, thereby preserving blood-brain barrier integrity (48). The second model involves AS01-associated trained innate immunity and systemic immune remodeling (14, 57). Trained immunity is a form of innate immune memory in which epigenetic and metabolic reprogramming alters the response of innate immune cells to subsequent challenges without relying on antigen-specific receptors (58). A randomized translational trial demonstrated that RZV—but not ZVL—induces durable, memory-like trained immunity in monocytes, natural killer cells, and dendritic cells that persists for up to 5 years post-vaccination. This training is driven by epigenetic modifications that persistently suppress transcription of the regulatory cytokine TGF-β-1, enhancing myeloid cell responsiveness to heterologous challenges (51). This adjuvant-mediated mechanism is epidemiologically supported by propensity-matched cohort studies showing that the AS01-adjuvanted RSV vaccine—which offers no protection against VZV—nonetheless extends dementia-free survival by an average of 87 days compared to influenza vaccination (**Figure 2B**) (16). RZV (Shingrix) used the full-dose AS01B formulation (50 μg monophosphoryl lipid A (MPL) and 50 μg purified saponin QS-21), while RSV vaccine (Arexvy) contains the dose-sparing AS01E formulation (25 μg MPL and 25 μg QS-21) (14, 15). Despite this difference in dose, both formulations have MPL selectively engages the TLR4-MyD88/TRIF signaling axis, while the purified saponin QS-21 promotes lysosomal destabilization and activates the NLRP3 inflammasome within antigen-presenting cells, promoting an interferon-gamma (IFN-γ) signaling cascade that drives microglial clearance of pathologically aggregated proteins (59, 60). Because these pathways likely interact *in vivo*, recombinant zoster vaccination may operate via a dual-pathway synergy—simultaneously suppressing viral reactivation and leveraging adjuvant-induced trained immunity to enhance neuroimmune homeostasis.

The latest evidences from natural experiments and large cohort studies links RZV to lower risks of cardiovascular events and stroke (61, 62). The natural experiment spanning the US transition from ZVL to RZV showed that RZV was associated with a 9% reduction in cardiovascular composite burden (RMTL ratio = 0.91, 95% Cl: 0.88 to 0.95), including reductions in ischemic heart disease and heart failure (62). In 2025, the similarly report showed that the two doses of RZV were associated with a 42.5% reduction in hospitalized stroke (aHR = 0.575) and a 28% reduction in acute myocardial infarction (aHR = 0.72) among adults aged over 50 years (61). These studies proposed that AS01-driven epigenetic and functional reprogramming of monocytes, including reduced IL-6 hyper-reactivity to TLR stimulation, may help stabilize the vascular endothelium (62).

Interactions between adaptive and innate immune compartments may help sustain AS01-induced trained immunity. In a randomized trial involving younger and older adults, found that RZV induced polyfunctional CD4+ T cells with predominant CD40L expression in both age groups (63). T-cell-derived CD40L can engage CD40-TRAF6 signaling in human monocytes and promote the epigenetic programming of durable trained immunity (63, 64). Proteomic profiling further showed that RZV reduced circulating levels of MCP-1, MCP-4, TNF, IL-18R1, and TWEAK in older adults after boost vaccination (63). This anti-inflammatory shift could help preserve blood-brain barrier integrity, reduce peripheral endotoxemia, and support homeostatic microglial clearance of abnormal amyloid and tau aggregates in the CNS.

Specifically, several pathways could connect the peripheral immune remodeling to cognitive outcomes. Enhanced monocyte and natural killer cell responsiveness may improve control of latent or recurrent infections, reducing repeated inflammatory and vascular insults (58). Changes in circulating cytokines and monocyte activity may also limit endothelial activation and help preserve blood-brain barrier function, thereby reducing the transmission of peripheral inflammatory signals to the CNS (65, 66). The cardiovascular and cerebrovascular associations reported after RZV are compatible with such a neurovascular pathway (61, 62), although they do not demonstrate that it mediates the observed association with dementia. In parallel, peripheral immune stimulation may induce innate immune memory in the brain, durably reshaping myeloid cell phenotypes and altering the handling of amyloid-β (Aβ), with potential consequences for the subsequent course of neuropathology (67).

Taken together, these findings support a model in which recombinant zoster vaccination may act through two complementary mechanisms suppression of subclinical viral reactivation and adjuvant-induced immune remodeling. Whether these mechanisms restore neurovascular and neuroimmune homeostasis, and how much each contributes to the observed associations, remains to be established.

## 6 Conclusion and future perspectives

The lifelong latency established by Varicella-Zoster Virus (VZV) in sensory neurons is not entirely quiescent, but rather represents a dynamic equilibrium maintained by continuous VZV-specific T-cell immunosurveillance. When systemic immunity wanes, this balance is disrupted, precipitating VZV reactivation—including asymptomatic episodes. Accumulating evidence from both *in vitro* models and *in vivo* phenotypes demonstrates that such reactivation triggers sustained, chronic neuroinflammation, which subsequently drives progressive central nervous system (CNS) dysfunction and the pathology of neurodegenerative disorders, particularly dementia.

Currently, epidemiological data spanning diverse study designs, data registries, and clinical settings provide a robust triangulation of evidence, consistently demonstrating that shingles vaccination—most notably the recombinant gE vaccine adjuvanted with AS01 (RZV)—is associated with a significantly reduced risk of dementia. Mechanistically, two non-mutually exclusive hypotheses have been proposed to explain this neuroprotective association: first, vaccine-induced antigen-specific immune responses may suppress subclinical VZV reactivation, thereby attenuating the downstream cascade of neuroinflammation; second, the AS01 adjuvant system, beyond bolstering multifunctional CD4+ T cell and antibody responses, may drive systemic immune remodeling to modulate CNS immune homeostasis, thereby enhancing the clearance of aberrant protein aggregates.

Although these epidemiological associations are consistent across diverse study designs, healthy-vaccinee selection bias and delays in clinical diagnosis cannot be fully excluded. Defining the role of VZV in chronic CNS neuropathology will require experimental studies through humanized non-human primate models of varicella reactivation and scalable platforms based on committed human sensory neurons. From a translational perspective, optimizing adult immunization strategies may have public health relevance, particularly because completing the full two-dose RZV regimen is associated with a greater dementia risk reduction compared to a single dose. Target trial emulations indicate that the protective benefits of shingles vaccination may extend across different stages of the disease course. Successfully preventing mild cognitive impairment and even slowing the rate of mortality among patients already living with dementia. ZVL vaccination has been associated with fewer diagnoses of mild cognitive impairment and lower mortality among people already living with dementia (56). Recent real-world studies have also compared ZVL and RZV vaccines administered after dementia diagnosis. ZVL primarily boosts antigen-specific T cell response, which wane over 3 to 5 years. In contrast, the AS01-adjuvanted RZV induces stronger and more durable cellular responses and may promote sustained innate immune training. Cohort analyses and target trial emulations, associate post-diagnosis RZV with better survival than ZVL (68).

Given the massive global burden of neurodegeneration, the initiation of large-scale, randomized controlled trials—such as the recently launched phase IV placebo-controlled trial of Shingrix in Finland enrolling 33,609 participants older than 76 years (NCT06436976). The trail is essential to prospectively confirm vaccine-mediated neuroprotection and guide its integration into preventative geriatric clinical frameworks.
